# The Texas State Medical Association

**Published:** 1886-11

**Authors:** 


### SOCIETY NOTES.

### THE TEXAS STATE MEDICAL ASSOCIATION.

(of especial interest to certain members.)

The growth and prosperity of this organization has been something remarkable, and has attracted the attention of the profession at large, not only at home but abroad. A few years ago it was scarcely known, outside the State, that there was such an organization; but within two years—since the Belton meeting, where it seems to have taken on a new impetus, we may say, in round numbers, that the membership has about doubled; and its work, as evidenced by the handsome volumes of yearly Transactions, will compare favorably with that of many older associations. The medical press has been exceedingly complimentary in speaking of it, and of the *esprit du corps* which has characterized the body; and to-day, in all parts of the United States, physicians, authors, statisticians, boards of health, insurance companies—all who are interested in such matters, properly regard the Association as the official, and a very creditable exponent of rational medicine in the great State of Texas. The spirit of organization, once aroused, has pervaded the land, and we hope, ere many years, to chronicle the enrollment of the entire regular profession of the State.

These facts should appeal to the pride of the members, individually, and stimulate them to greater exertions. It is not enough to be punctual in attendance, and to regularly contribute papers to the several sections—tho' both of these are recognized as self-evident duties; but each one should exert himself to induce others to join and to do likewise; should heartily co-operate in the endeavor to bring within the parent fold all legitimate practitioners in good standing within the State.

Moreover, they should be scrupulously exact, and prompt to pay their *pro rata* share of the expense incidental to carrying on the work. It is not at all creditable to the Association, that some sixty odd members allowed their names to be dropped from the roll for non-payment of yearly dues; and now that a resolution appears on the books permitting those members to be reinstated on the same terms that new members are admitted, we sincerely trust that they will hasten to do so.

The direct object of this article is to call attention to the fact that there are others than those already dropped who are in arrears, and who will surely be suspended unless they heed the warning. The Secretary, by direction of the President, sent out in August, a circular letter to delinquents, setting forth the fact that the Treasury would be (and now is) pretty well depleted by the heavy expense,—partly of publishing a large volume of Transactions—and calling on them to pay up. We are sorry to say that up to date *very few* have done so,—notwithstanding each one was notified of the amount due, and of the *urgent need for its immediate use.*

As others than members will read this, we will not go into details; suffice it to say that the Association *is in need of the money*, and if there are any who do not know how their account stands, the information will be furnished by the affable and efficient Treasurer, Dr. J. Larendon, at Houston, to whom all remittances should be made, *and not to the Secretary.*

The handsome volume of transactions has been mailed to the members. To those who have not yet responded to the President's call, we trust it will serve as a forcible reminder of *an imperative duty.*

